# Examining the safety of respiratory and intravenous inoculation of *Bdellovibrio bacteriovorus* and *Micavibrio aeruginosavorus* in a mouse model

**DOI:** 10.1038/srep12899

**Published:** 2015-08-07

**Authors:** Kenneth Shatzkes, Richard Chae, Chi Tang, Gregory C. Ramirez, Somdatta Mukherjee, Liana Tsenova, Nancy D. Connell, Daniel E. Kadouri

**Affiliations:** 1 Division of Infectious Disease, Department of Medicine, Rutgers New Jersey Medical School, Newark, NJ 07103, USA; 2 Department of Oral Biology, Rutgers School of Dental Medicine, Newark, NJ 07103, USA; 3 Laboratory of Mycobacterial Immunity and Pathogenesis, Public Health Research Institute, Rutgers New Jersey Medical School, Newark, NJ 07103, USA; 4 Department of Biological Sciences, New York City College of Technology, Brooklyn, NY 11201, USA

## Abstract

*Bdellovibrio* spp. and *Micavibrio* spp. are Gram-negative predators that feed on other Gram-negative bacteria, making predatory bacteria potential alternatives to antibiotics for treating multi-drug resistant infections. While the ability of predatory bacteria to control bacterial infections *in vitro* is well documented, the *in vivo* effect of predators on a living host has yet to be extensively examined. In this study, respiratory and intravenous inoculations were used to determine the effects of predatory bacteria in mice. We found no reduction in mouse viability after intranasal or intravenous inoculation of *B. bacteriovorus* 109J, HD100 or *M. aeruginosavorus.* Introducing predators into the respiratory tract of mice provoked a modest inflammatory response at 1 hour post-exposure, but was not sustained at 24 hours, as measured by RT-qPCR and ELISA. Intravenous injection caused an increase of IL-6 in the kidney and spleen, TNF in the liver and CXCL-1/KC in the blood at 3 hours post-exposure, returning to baseline levels by 18 hours. Histological analysis of tissues showed no pathological changes due to predatory bacteria. Furthermore, qPCR detected predators were cleared from the host quickly and efficiently. This work addresses some of the safety concerns regarding the potential use of predatory bacteria as a live antibiotic.

*Bdellovibrio bacteriovorus* and *Micavibrio aeruginosavorus* are small, highly motile, uniflagellate Gram-negative bacteria that prey naturally on other Gram-negative bacteria^1^,^2^. Recently, predatory bacteria have been considered as potential alternatives to traditional antibiotics for treating multi-drug resistant (MDR) Gram-negative bacterial infections. *B. bacteriovorus* have a predatory lifestyle where they attach to and enter the prey periplasm, multiply by exhausting the nutrients, lyse the cell, and then continue to seek out more prey to invade^1^,^3^,^4^. *Micavibrio* spp., in contrast, attach to, grow and kill prey at the surface of the prey cell in a ‘vampire’-like fashion^2^,^5^,^6^.

*Bdellovibrio*-and-like organisms (BALOs) are a promising potential novel agent against bacterial pathogens and present several advantages when considering their use for controlling infection^7^. Previous studies have confirmed the ability of predatory bacteria to control a broad range of important human pathogens *in vitro*, including MDR bacteria^8^, grown both planktonically and in biofilms^9^,^10^,^11^. In addition, BALOs appear to have no negative effect on human cells when challenged *in vitro*^12^. Recent studies have presented evidence that BALOs might be native commensals of the human gut and might even play a role in maintaining healthy gut flora^13^. In addition, development of genetically stable predation-resistance in a normally susceptible species has yet to be confirmed^14^, a major advantage over current available antibiotic therapies. To date, the majority of the studies dealing with predatory bacteria have been performed *in vitro*; the *in vivo* effect of predatory bacteria in a mammalian host is still not well understood.

Early animal studies found *B*. *bacteriovorus* to be non-pathogenic when injected into mice, rabbits and guinea pigs^7^,^15^, while another study demonstrated that *B*. *bacteriovorus* could not survive in the gastrointestinal tracts of fish, mice and frogs^16^. A more recent study showed predatory bacteria are non-toxic when fed to young chicks^17^. To our knowledge, these studies were limited to observation of the animal host, with no examination of the host immunological response to predatory bacteria inoculation *in vivo*.

Before predatory bacteria can be used clinically, their safety in a mammalian host must be confirmed. In this study, respiratory and intravenous inoculation mouse models were used to demonstrate the effects of predatory bacteria. The work presented here highlights the potential use of predatory bacteria as a future biological-based agent for controlling infection.

## Results

### Effect of Respiratory Introduction of Predatory Bacteria

#### Host viability and histology

To examine the effect of respiratory exposure of predatory bacteria on host survival, we administered intranasally 1 × 10^9^ PFU/mouse of *B. bacteriovorus* 109J, HD100 or 1 × 10^6^ PFU/mouse of *M. aeruginosavorus* ARL-13 to three groups of C57BL/6 mice (5 mice per group) and monitored animals for any signs of infection, illness or discomfort. To measure the effect of predatory bacteria cell particles, 1 × 10^9^ PFU/mouse of non-viable heat-killed *B. bacteriovorus* 109J or HD100 were also administered to two other groups of mice. Phosphate buffered saline (PBS) was used as a negative control. At five days post-inoculation, all 25 mice inoculated with viable or heat-killed predatory bacteria were healthy with no visual adverse effects or change in behavior (Table 1). At this point, three mice were sacrificed for further evaluation and two mice from each group were kept and visually assessed for up to 50 days. As before, no visual signs of infection were seen, with all inoculated mice remaining viable and healthy 50 days post-inoculation (Table 1).

**Figure 1:**
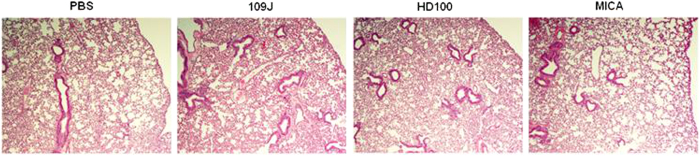
Histological examination of mouse lungs after respiratory introduction of predatory bacteria. Mice were inoculated intranasally with *B. bacteriovorus* 109J, HD100 or *M. aeruginosavorus* ARL-13. Histological examination of lungs exposed to *B. bacteriovorus* and *M. aeruginosavorus* revealed no pathology compared to the control mice treated with PBS. All images are representative micrographs that were taken at 48 hours post-intranasal inoculation and at 40X total magnification.

Forty-eight hours post-inoculation, histological examination of the lungs and spleens of mice inoculated with *Bdellovibrio* or *Micavibrio* revealed no pathology compared to the control mice, treated with PBS (Fig. 1). Lung parenchyma showed normal appearance and was well preserved in most of the sections. Some of the sections from both groups (inoculated and control) showed increased cellularity in some areas, predominantly mononuclear cells (lymphocytes and macrophages), but no neutrophils. It is most likely these changes resulted from removing and processing the tissue.

### Host immune response to predatory bacteria

To examine the effects of introduction of predatory bacteria via the respiratory tract on the host immune response, we introduced each predator through intranasal inoculation into the respiratory tract of mice. Mice were visually monitored for signs of illness or discomfort, and euthanized at 1, 24 or 48 hours post-inoculation when organs and blood were harvested.

For the one hour time point experiment, 6 mice per predatory bacterial strain were exposed to 4 × 10^9^ PFU/mouse of *B*. *bacteriovorus* 109J, 7 × 10^9^ PFU/mouse of *B*. *bacteriovorus* HD100 or 5 × 10^8^ PFU/mouse of *M*. *aeruginosavorus* ARL-13. At the 24 and 48 hour time points, 12 mice per strain were exposed to an inoculation dose of 1 × 10^9^ PFU/mouse of both *B*. *bacteriovorus* 109J, HD100 or 1 × 10^11^ PFU/mouse of *M*. *aeruginosavorus* ARL-13. Total RNA was extracted from the lungs and spleen, and expression of inflammatory cytokines was measured using RT-qPCR.

**Figure 2:**
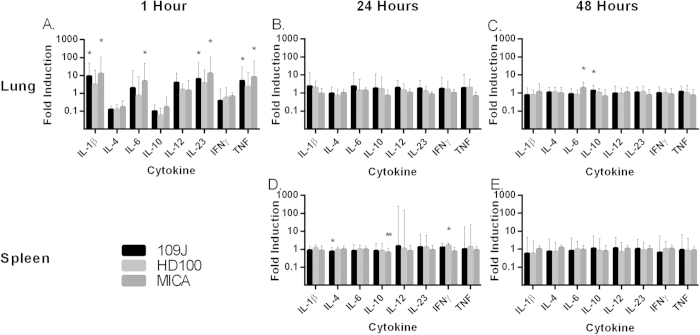
Inflammatory cytokine profile in response to respiratory introduction of predatory bacteria. qPCR analysis of IL-1β, IL-4, IL-6, IL-10, IL-12, IL-23, IFNγ, and TNF in response to intranasal inoculation of predatory bacteria relative to PBS control. Mice were inoculated intranasally with *B. bacteriovorus* 109J, HD100 or *M. aeruginosavorus* ARL-13. Expression of cytokines was assessed in the lung at (**A**) 1, (**B**) 24, and (**C**) 48 hours post-inoculation. Expression of cytokines was assessed in the spleen at (**D**) 24 and (**E**) 48 hours post-inoculation. Fold induction was calculated using the ΔΔC_t_ by approximation method using an endogenous calibrator (β-actin). For the one hour experiment, 6 mice per predatory bacterial strain (and PBS) were used. Twelve mice per strain (and PBS) were used at each of the 24 and 48 hour time points, with the exception of the Lung/24 hour experiment, where 6 mice were used. Data for the one hour time point is from one experiment; data for the 24 and 48 hour time points are from two independent experiments. Data represent mean ± standard deviation. Student’s *t*-test was performed to compare each treated sample to their respective control sample, *P < 0.05; **P < 0.01.

**Figure 3:**
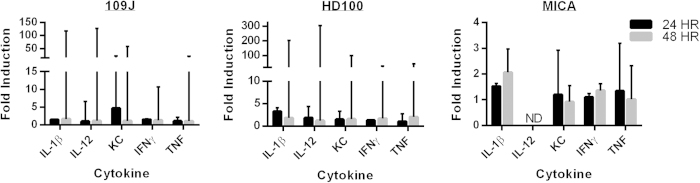
Inflammatory protein profile of the lung in response to intranasal inoculation of predatory bacteria. ELISA analysis of IL-1β, IL-12, CXCL-1/KC, IFNγ, and TNF in response to intranasal inoculation of predatory bacteria relative to PBS control. Mice were inoculated intranasally with *B. bacteriovorus* 109J, HD100 or *M. aeruginosavorus* ARL-13. Inflammatory proteins were assessed in the lung at 24 and 48 hours post-inoculation of (**A**) *B. bacteriovorus* 109J, (**B**) HD100, and (**C**) *M. aeruginosavorus* strain ARL-13. 12 mice per treatment group were used at each time point. Data from two independent experiments. Data represent mean ± standard error of the mean. Student’s *t*-test was performed to compare each treated sample to their respective control sample, *P < 0.05; **P < 0.01.

As before, none of the total 90 mice that were inoculated with predatory bacteria exhibited any visual adverse effects and all were healthy at the time of sacrifice. At one hour post-inoculation, we observed an increase of IL-1β (9.0- and 12.3-fold), IL-23 (6.3- and 12.6-fold) and TNF (5.0- and 7.9-fold) in the lungs of mice exposed to *B. bacteriovorus* 109J or *M. aeruginosavorus*, respectively (Fig. 2A). However, this increased expression was not sustained at 24 or 48 hours post-inoculation (Fig. 2B,C). Conversely, none of the mice exposed to *B. bacteriovorus* HD100 exhibited a substantial (5-fold or higher) increase in expression of any inflammatory marker gene in the lung or spleen at any time point (Fig. 2). Furthermore, no inflammatory response was detected in the spleens of mice inoculated with either *B. bacteriovorus* 109J or *M. aeruginosavorus* at 24 or 48 hours (Fig. 2D,E). Inflammatory protein levels in the lungs of inoculated mice were measured with ELISA to confirm the results obtained from qPCR. We did observe a 4.7-fold increase in CXCL-1/KC protein expression due to *B. bacteriovorus* 109J at 24 hours. However, no other inflammatory protein showed a substantial fold induction (5-fold or higher) due to inoculation with any of the predatory bacteria (Fig. 3). Additionally, mice examined at five days post-inoculation still exhibited no substantial increases in proinflammatory marker gene expression (data not shown). As before, all mice inoculated with the predators were healthy with no visual adverse effects at any of the examined time points.

To assess the change in cytokine levels in the host’s response to a known respiratory bacterial pathogen, we introduced 1.2 × 10^9^ CFU/mouse of *K. pneumoniae* to mice (n = 2) through intranasal inoculation. As expected, we observed a 2260- and 80-fold induction of IL-6 in the lungs and spleen, respectively, as well as a 21-fold induction of TNF in the lungs of the host at 24 hours post-infection. Our data are consistent with previously published studies that demonstrate *K. pneumoniae* to elicit a strong cytokine response in the mouse lung^18^,^19^. In comparison, mice exposed to *B. bacteriovorus* 109J or *M. aeruginosavorus* showed only a 1.9- and 4.7-fold induction of IL-6, respectively at 1 hour post-inoculation (Fig. 2A), reflecting a much stronger immune response to *K. pneumoniae*. Collectively, our data indicate that when inhaled, predatory bacteria do not provoke an elevated, sustained immune response in mice.

### Effect of Intravenous Introduction of Predatory Bacteria

#### Host viability and histology

**Figure 4:**
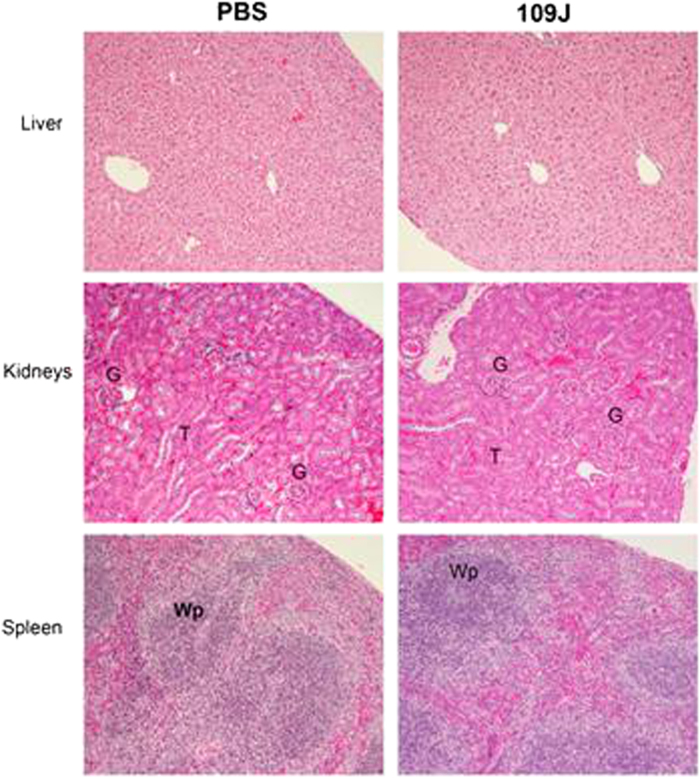
Histological examination of mice after intravenous injection of *B. bacteriovorus* 109J. Histological examination of mice injected through the tail vein with *B. bacteriovorus* 109J revealed no pathology compared to the control mice treated with PBS. All images are representative micrographs that were taken at 20 days post-tail vein injection and at 100X total magnification. G–glomeruli; T–tubules; Wp–white pulp.

The effect of predatory bacteria introduced via the intravenous route was also investigated. To this end, 1 × 10^8^ PFU/mouse of *B*. *bacteriovorus* 109J or PBS control were injected into the tail vein of mice (5 mice per group). At 20-days post-inoculation, all mice injected with predatory bacteria were viable and healthy (Table 2). To model a multiple bacteremia event, a group of 5 mice were re-injected with 1 × 10^8^ PFU/mouse of *B*. *bacteriovorus* 109J at 10-days post-initial injection. Again, we did not observe any reduction in mouse viability due to re-injection of predatory bacteria (Table 2). Histological examination, taken 20 days following injection, of the liver, kidney, and spleen revealed no pathology or signs of inflammation compared to the control mice inoculated with PBS (Fig. 4). Micrographs of the liver showed normal hepatic cells in both predator-infected mice and unexposed controls. Kidneys also showed normal structure with glomeruli and tubules. No pathology was detected in the spleen, with well-preserved red (presence of erythrocytes) and white (tightly packed lymphocytes) pulp.

### Blood profiling

**Figure 5:**
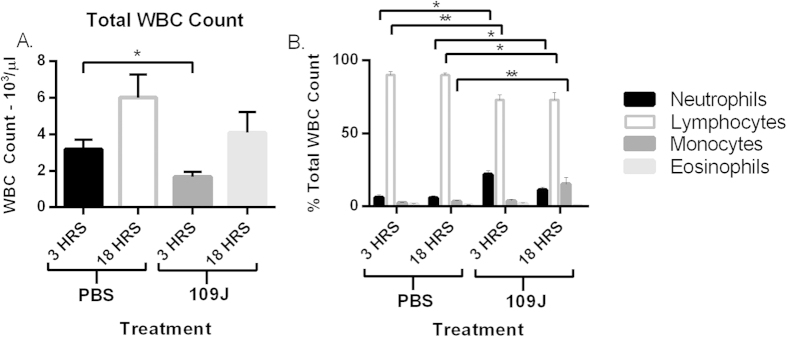
Inflammatory cell response to intravenous injection of *B. bacteriovorus* 109J. To profile the host cell response in the blood, mice were injected through the tail vein with *B. bacteriovorus* 109J. Profile of (**A**) total white blood cell (WBC) counts and (**B**) inflammatory cells after tail vein injection of *B. bacteriovorus* 109J. Blood was assessed at 3 and 18 hours post-injection. Blood profiles were performed by ANTECH Diagnostics (New Hyde Park, NY, USA). Data represent mean ± standard error of the mean. Student’s *t*-test was performed to compare each treated sample to their respective control sample, *P < 0.05; **P < 0.01.

To determine the effect of intravenous inoculation of predatory bacteria on host blood cell profile, 100 μl of blood was removed from each mouse at 3 and 18 hours post-exposure. White blood cell (WBC) counts were performed and the levels of individual cell types determined (Fig. 5). Surprisingly, total WBC counts decreased at 3 and 18 hours post-injection compared to the control. A 3.5- fold increase in the percentage of neutrophils in the blood was seen at 3 hours post-injection in mice inoculated with *B. bacteriovorus* 109J. However, the level of neutrophils in the blood returned to comparable levels seen in control animals by 18 hours post-exposure. In contrast, the percentage of monocytes in the blood remained elevated by 4.7-fold at 18 hours post-injection. Decreased percentages of lymphocytes in the blood were seen in conjunction with the observed increases of neutrophils and monocytes resulting from inoculation with predatory bacteria. Eosinophils were found at comparably low levels in both the control and treated mice.

### Host immune response to B. bacteriovorus 109J

**Figure 6:**
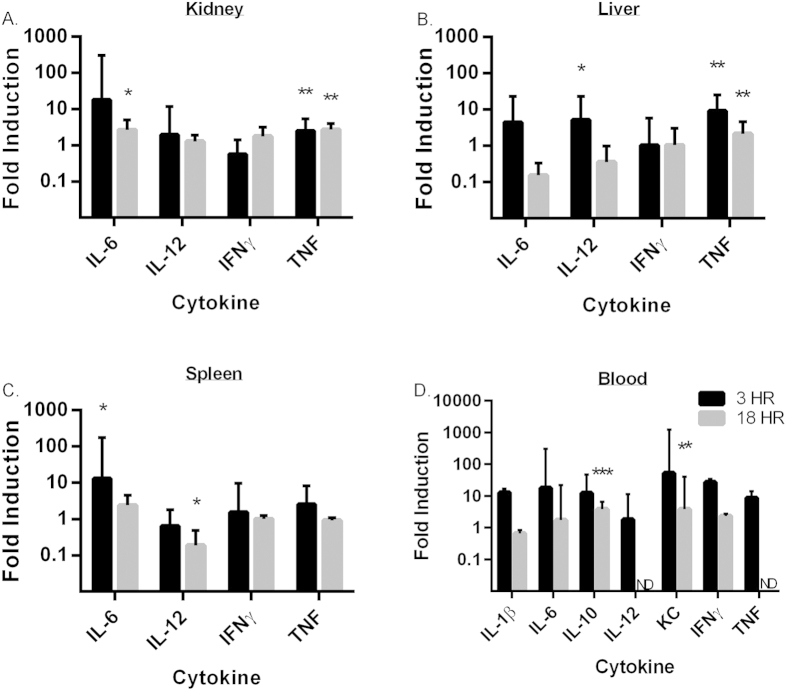
Inflammatory cytokine profile in response to intravenous injection of *B. bacteriovorus* 109J. (**A**–**C**) qPCR analysis of IL-6, IL-12, IFNγ, and TNF in response to tail vein injection of *B. bacteriovorus* 109J relative to PBS control. Expression of cytokines was assessed in the (**A**) kidney, (**B**) liver and (**C**) spleen at 3 and 18 hours post-injection. Fold induction was calculated using the ΔΔC_t_ by approximation method using an endogenous calibrator (β-actin). Five mice per treatment group were used at each time point. Data represent mean ± standard deviation. (**D**) ELISA analysis of IL-1β, IL-6, IL-10, IL-12, CXCL-1/KC, IFNγ, and TNF in response to tail vein injection of *B. bacteriovorus* 109J relative to PBS control. Inflammatory proteins were assessed in the blood at 3 and 18 hours post-injection. Five mice per treatment group were used at each time point. Data represent mean ± standard error of the mean. Student’s *t*-test was performed to compare each treated sample to their respective control sample, *P < 0.05; **P < 0.01; ***P < 0.001.

To examine the effects of intravenous introduction of predatory bacteria on the host immune system, we injected 1 × 10^8^ PFU/mouse of *B*. *bacteriovorus* 109J into the tail vein of mice (5 mice per treatment group). Mice were visually monitored for signs of illness or discomfort, and euthanized at either 3, 18 hours or 20 days post-injection, when organs and blood were harvested. To model a multiple bacteremia event, a group of mice were re-injected with 1 × 10^8^ PFU/mouse of *B*. *bacteriovorus* 109J at 10 days post- initial injection. The kidney, liver and spleen were harvested to measure inflammatory cytokines (Fig. 6).

As we observed in the respiratory inoculation model, none of the 20 mice that were injected with predatory bacteria exhibited any observable adverse effects. At 3 hours post injection, we detected an increase in inflammatory cytokines TNF (9.0-fold) in the liver (Fig. 6B) and IL-6 (18- and 13-fold) in the kidney and spleen (Fig. 6A,C, respectively) relative to control. However, as with our results obtained from respiratory introduction, this increased expression was not sustained by 18 hours post-injection (Fig. 6). An ELISA using whole blood from inoculated mice revealed increases in inflammatory proteins, including IL-1β (13-fold), IL-6 (18-fold), IL-10 (13-fold), CXCL-1/KC (53-fold), IFNγ (27-fold) and TNF (8.7-fold), at 3 hours post-injection, but returned to baseline levels by 18 hours post-injection (Fig. 6D). Taken together, the data suggest that intravenous injection of *B. bacteriovorus* 109J does not provoke a sustained inflammatory response. Our data reflect the host’s response to and efficient clearance of the invading organism.

### Bacterial Dissemination within the Host

**Figure 7:**
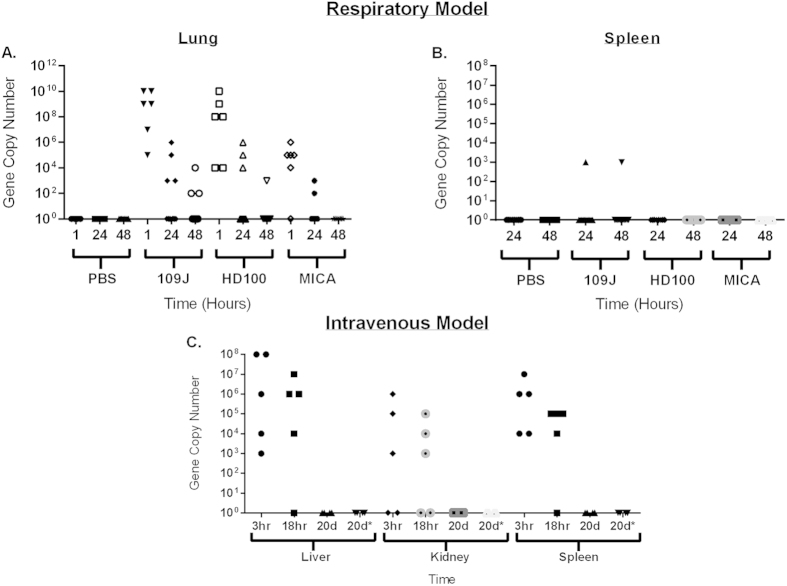
Predatory bacterial dissemination within host. qPCR detection of predatory bacteria within the host. For the respiratory model, the (**A**) lung and (**B**) spleen were probed for *B. bacteriovorus* 109J, HD100, and *M. aeruginosavorus*. In the intravenous model (**C**), the liver, kidney and spleen were probed for only *B. bacteriovorus* 109J. In the respiratory model, 6 mice per predatory bacterial strain (and PBS) were used at the one hour time point; 12 mice per treatment group were used at the 24 and 48 hour time points. Five mice per treatment group at each time point were used in the intravenous model. Each data point represents a single mouse’s bacterial load. 20d* - mice re-injected with *B. bacteriovorus* 109J or PBS control at 10 days post-initial-injection (to model multiple bacteremia event).

In order to examine predatory bacterial dissemination and migration following inoculation, we utilized primers targeting the 16S ribosomal RNA region for each of *B. bacteriovorus* 109J, HD100, or *M*. *aeruginosavorus*. Total RNA from organ samples collected from the previously described respiratory and intravenous mouse experiments were probed for detectable levels of predators using RT-qPCR (Fig. 7). For the one hour time point, an inoculation dose of 4 × 10^9^ PFU/mouse of *B*. *bacteriovorus* 109J, 7 × 10^9^ PFU/mouse of *B*. *bacteriovorus* HD100 or 5 × 10^8^ PFU/mouse of *M*. *aeruginosavorus* ARL-13 was used. For each of the 24 and 48 hour time points, an inoculation dose of 1 × 10^9^ PFU/mouse of both *B*. *bacteriovorus* 109J or HD100, or 1 × 10^11^ PFU/mouse of *M*. *aeruginosavorus* ARL-13 was introduced.

In the respiratory model, at one hour post-inoculation, *B. bacteriovorus* 109J was detected in the lungs in 6 out of the 6 mice examined (ranging from 10^5^ to 10^10^ gene copy numbers), HD100 in 6/6 mice (10^4^–10^10^), and *M*. *aeruginosavorus* in 5/6 mice (10^4^–10^6^), (Fig. 7A). However, the number of predatory bacteria detected in the lungs dropped substantially by 24 and 48 hours post-inoculation with all tested strains. No predators were detected in the spleens of mice at either 24 or 48 hours post-inoculation (Fig. 7B).

In the intravenous model, *B. bacteriovorus* 109J was detected at high levels (10^3^–10^8^) in 5 out of the 5 injected mice at 3 hours post-injection (Fig. 7C). A modest drop in detectable 109J was seen at 18 hours post-injection, with complete clearance of the predators in all mice observed at 20 days post-injection. In addition, no predators were detected 10 days post-second injection in the multiple bacteremia model, suggesting complete clearance of the predators by 10 days post-injection (Fig. 7C). Altogether, our data indicate that *B. bacteriovorus* 109J bacteria are quickly and efficiently cleared from the tissue of mice exposed either intranasally or intravenously.

## Discussion

The antibiotic-resistance crisis has inspired researchers in recent years to look for new approaches to treat life-threatening bacterial infections. One biologically-based microbial control strategy is the use of predatory bacteria^7^. The ability of predatory bacteria to prey efficiently on Gram-negative bacteria suggests a promising, novel way to combat infection. However, while efficacy has been shown *in vitro*, the effects of predatory bacteria *in vivo* have not been extensively examined.

In this study, we assessed the effect of predatory bacteria exposure in the mouse. To verify that the results are not strain-specific, we used two strains of *B. bacteriovorus*; we are not aware of additional *M. aeruginosavorus* strains other than the ARL-13 strain. We administered high doses of predatory bacteria to mice via the respiratory and intravenous routes and examined the effect on host viability and immune response. Across the entire study, a total of 105 mice were inoculated intranasally and 20 mice were intravenously injected with predatory bacteria. In both models, we observed neither reduction in host viability nor adverse effects when administering high concentrations of predatory bacteria. A multiple bacteremia model also showed no effect on mouse viability after repeat exposure to predators. Furthermore, histological examination of tissue revealed no pathology in any of the organs tested, suggesting predatory bacteria have no visible negative effects on the overall health of the mice. To reduce the number of animals being sacrificed in the study, only *B. bacteriovorus* 109J was used in the intravenous model. Future studies should involve additional isolates to confirm the results.

Our results align with the findings reported in other animal models which found predatory bacteria non-toxic^15^,^16^,^17^. One such study evaluated the effects of *B. bacteriovorus* HD100 when orally administered to young chicks^17^. Surprisingly, *B. bacteriovorus* HD100 was found to be adaptable and was able to survive in the anaerobic conditions and higher body temperatures of the chick gut. While oral administration of the predators altered the chick’s normal gut microbiota, there were no other visual adverse effects on their well-being. However, the study did not assess the chick’s immune response to predatory bacteria, and combined with the lack of adverse effects on the host, this left questions as to the immunogenicity of predatory bacteria in a living host.

We next looked to profile the host immune response to predatory bacteria introduction. We detected a modest immune response to predatory bacteria in both the respiratory and intravenous models. An increase in specific proinflammatory cytokines and chemokines was detected (namely IL-1β, IL-6, IL-23, CXCL-1/KC, IFNγ, and TNF). However, the response paled in comparison to the response caused by a known respiratory bacterial pathogen, *K. pneumoniae*. The initial increase in proinflammatory cytokines was not sustained, and cytokine levels were back to baseline levels by 24 and 18 hours post-inoculation, for the respiratory and intravenous models, respectively. Furthermore, bacterial dissemination experiments showed predatory bacteria were efficiently cleared from the host in both models. Although all mice were initially inoculated intranasally with 4 × 10^9^ PFU/mouse, qPCR was able to detect 10^10^ gene copies of *Bdellovibrio* in three of the mice. qPCR has been found to slightly overestimate quantities of bacteria compared to standard microbiological plating methods, as there is no discrimination in amplification between viable and dead cells^20^; this could account for the slight difference in the numbers we observed. We detected no predators in the spleen at either 24 or 48 hours, possibly due to the predators being cleared from the host before reaching the spleen. The intravenous model showed complete clearance of the predators by 20 days post-injection and also determined that *B. bacteriovorus* 109J bacteria inoculated by repeated injections were just as efficiently cleared.

The proinflammatory cytokines that were induced by exposure to predatory bacteria represent hallmarks of activation of the innate immune response. Furthermore, profiling of mice exposed to *B. bacteriovorus* 109J saw a 3.5-fold increase in the percentage of neutrophils present in the blood at 3 hours post-injection. Neutrophils are key players in the innate immune response and constitute the first line of defense against invading pathogens^21^. The increase in neutrophils in the blood correlates with the large increase of CXCL-1/KC (53-fold) and IFNγ (27-fold) expression in the blood at 3 hours, as analyzed through ELISA. CXCL-1 is expressed by macrophages, neutrophils and epithelial cells. Both CXCL-1 and IFNγ have been found to have neutrophil chemoattractant activity^22^,^23^. Thus, we suspect that *B. bacteriovorus* 109J is being cleared from the blood by neutrophils recruited to the site through a chemotactic gradient of expressed cytokines and chemokines.

While this limited immune response to predatory bacteria exposure may surprise some, it is important to note that predatory bacteria may be inherently non-pathogenic to mammalian hosts. A study looking at the effects of non-pathogenic Gram-negative bacteria on the immune response in the gut found similar patterns in cytokine expression levels when challenging with non-pathogenic strains of *Escherichia coli*, as well as an increase in TLR-4 expression^24^. Toll like receptors are a family of pattern recognition receptors that play a key role in innate immunity. It has been reported that *B. bacteriovorus* contains a unique lipopolysaccharide (LPS) possessing neutral lipid As containing α-D-mannoses that replace the usual negatively-charged phosphate residues found in the LPS on pathogenic bacteria^25^. The same study showed that this unique LPS provokes a weak immunogenic response from human mononuclear cells *in vitro*. Similar to our observations in our *in vivo* model, they detected smaller inductions of TNF and IL-6 as compared to that induced by pathogenic *E*. *coli*. As TLR-4 is responsible for detecting LPS expressed on or released from the surface of Gram-negative bacteria to activate the innate immune response, the neutral-charged LPS on *B. bacteriovorus* prevents a more robust response and thus results in less inflammation. This may also explain the slightly larger induction of cytokines observed in the lungs when inoculating the host with *M*. *aeruginosavorus* as compared to the two *B. bacteriovorus* strains. The LPS of *M. aeruginosavorus* has not been characterized. However, a previous study focusing on the use of predatory bacteria to control ocular infections found that *B. bacteriovorus* and *M. aeruginosavorus* both induced weak expression of IL-8 and TNF in human corneal-limbal epithelial cells *in vitro*^12^, signaling that *M. aeruginosavorus* may contain an altered LPS as well. Further analysis of the LPS of *M. aeruginosavorus* must be done to confirm these results.

In conclusion, our results demonstrate that predatory bacteria *B*. *bacteriovorus* 109J, HD100 and *M*. *aeruginosavorus* ARL-13 are non-pathogenic in a mammalian host, do not induce a robust or sustained immune response, and are efficiently cleared from the host. Future studies should focus on assessing the efficacy of predatory bacteria to prey on Gram-negative pathogens *in vivo*.

## Methods

### Bacteria, strains and growth conditions

The predatory bacteria used in this study were *Bdellovibrio bacteriovorus* 109J (ATCC 43826), *B. bacteriovorus* HD100^3^ and *Micavibrio aeruginosavorus* strain ARL-13^6^. *Klebsiella pneumoniae* ATCC 43816 was used and grown in LB media. Predatory bacteria were cultured and processed as previously described^9^,^12^. Predator stock-lysates were prepared by co-culturing the predators with host cells in diluted nutrient broth (DNB) (a 1:10 dilution of nutrient broth supplemented with 3 mM MgCl_2_ and 2 mM CaCl_2_). The co-cultures were incubated at 30^°^C until the culture cleared (stock-lysates). To cultivate high concentrations of *Bdellovibrio* for inoculation experiments, 10 ml of washed overnight culture host cells (∼1 × 10^9^ CFU/ml) were re-suspended in 90 ml of DNB containing 10 ml of predatory bacteria from the stock-lysates. *Micavibrio* was initially cultured in the same manner. To obtain higher predator concentrations, *Micavibrio* co-cultures were prepared in 200 ml of DNB containing 20 ml of the host and 20 ml of *Micavibrio* stock-lysates. *Bdellovibrio* were incubated on a rotary shaker for 24 hours. *Micavibrio* were incubated on a rotary shaker for 48 hours for the initial experiment. After the initial experiment, a 72 hour incubation period was used to reach higher concentrations of the predator. To purify and concentrate the predators, co-cultures were passed three times through a 0.45-μm Millex pore-size filter (Millipore) to remove residual prey and cell debris (filtered lysate). To further purify and concentrate predator samples, filtered lysate was pelleted three times by centrifugation at 29,000 g for 45 min using a Sorvall LYNX 4000 centrifuge (Thermo Fisher Scientific Inc). Each time, the pellet was washed and re- suspended in 50 ml of phosphate buffered saline (PBS). For the last wash, the predator pellet was re-suspended in 1-2 ml of PBS solution to reach a final optical absorbance of ∼0.3–0.4 at 600 nm. Predator cell concentrations were determined each time using the standard double-layered agar method^26^. To confirm that the samples were free of host cells, 50 μl aliquots of the predator samples were removed and plated on LB agar TSB-blood plates. Since the predatory bacteria were used directly after isolation, the actual viable predator dose was known only a few days after each experiment, as the plaque-forming unit (PFU) appeared. Therefore, in some experiments, mainly involving *M. aeruginosavorus,* the inoculation size varied somewhat. Actual predator inoculation doses are specified for each experiment.

### Mice

Wild type C57BL/6 mice were purchased from the National Cancer Institute (Frederick, MD, USA). The mice were housed under pathogen-free conditions at the Rutgers New Jersey Medical School animal facility. All experiments were performed in accordance with the protocols approved by the Institutional Animal Care and Use Committee of Rutgers New Jersey Medical School (protocol #13112A1) and the Animal Care and Use Review Office of the U.S. Army Medical Research and Material Command.

### Respiratory inoculation model

Predatory bacteria were introduced by intranasal inoculation of C57BL/6 mice to model a respiratory infection. Animals were lightly anaesthetized with 3% isoflurane in oxygen for four minutes using an isoflurane vaporizer. Twenty-five μl of purified bacterial suspension were gently applied at both nostrils. Mice were inoculated with either PBS, *B. bacteriovorus* strain 109J, *B. bacteriovorus* strain HD100, or *M. aeruginosavorus* strain ARL-13. After initial inoculation, animals were observed for the following five days and visually assessed for signs of infection, illness and discomfort. Two mice from each treatment group were kept and visually assessed for up to 50 days. To assess the immune response, lung, serum, liver, and spleen samples were collected at 1, 24, and 48 hours post-exposure. Organs for RNA extraction were stored in 1.0 ml of Trizol at −80 °C. Organs for ELISA were stored at −80 °C in 1.0 ml of PBS containing protease inhibitor. Samples for histology were stored in 1.0 ml of paraformaldehyde at 4 °C.

### Intravenous inoculation model

Twenty-five μl of purified *B. bacteriovorus* strain 109J were introduced by tail vein injection to evaluate the effects of an acute bacteremia event on mouse viability and predator clearance. C57BL/6 mice were injected with either PBS or 1 × 10^8^ PFU/mouse *B. bacteriovorus* strain 109J. After initial inoculation, animals were observed for up to 20 days and visually assessed for signs of infection, illness and discomfort. To model a multiple bacteremia event, groups of mice were re-injected with either PBS or 1 × 10^8^ PFU/mouse *B. bacteriovorus* strain 109J at 10 days post-initial injection. To assess the host immune response, mice were kept for 3 or 18 hours post-exposure, when they were euthanized and lung, serum, liver, kidney and spleen samples collected.

### RNA extraction

Samples were prepared as previously described^27^. Organs were homogenized in bead beater tubes. To extract total RNA, liquefied samples were centrifuged at 13,200 RPM for 20 minutes at 4 °C to remove tissue debris, and the supernatants were transferred to a new tube. Two-hundred μl of chloroform were added, and centrifuged again at 11,600 RPM for 15 minutes. The aqueous phase was transferred to a new tube and combined with equal volume of isopropanol to precipitate the RNA. Samples were then centrifuged at 11,600 RPM for 15 minutes, and remaining isopropanol removed. Pellets were washed twice with 500 ml of ice-cold 70% ethanol, removing the ethanol from the sample after centrifugation. The pellets were then re-suspended in 30 μl of nuclease-free water. Total RNA was then purified using the ‘RNA Cleanup’ protocol in the RNeasy® Mini Kit (Qiagen), and stored at −80 °C.

### Host immune response profiling (qPCR)

Samples were prepared as previously described^27^. cDNA synthesis was performed on total RNA isolated using the High-Capacity RNA-to-cDNA™ Kit (Applied Biosystems) according to manufacturer’s instructions. To profile the host immune response, TaqMan® probes targeting selected cytokines and an endogenous calibrator (β-actin) were utilized for qPCR. Samples were tested in duplicate, with each reaction containing: template (2.0 μl of synthesized cDNA), TaqMan® Gene Expression Master Mix (Applied Biosystems), TaqMan® probe for selected cytokine (Applied Biosystems), and nuclease-free water to 10 μl. A 7900HT Fast Real-Time PCR System (Applied Biosystems) was used with the following protocol: 50 °C for 2 min (1X), 95 °C for 10 min (1X), 95 °C for 15 sec/60 °C for 1 min (40X). Relative quantification of cytokines was performed using the ΔΔC_t_ by approximation method (As described in Reference^28^ and^29^). Relative fold expression compared to control was calculated as 2^−ΔΔCt^, where ΔC_t_ = C_t_ (gene of interest)—C_t_ (normalizer = β-actin) and the ΔΔC_t_ = ΔC_t_ (sample)—ΔC_t_ (calibrator). Calibrator was total RNA from mice inoculated with PBS.

### ELISA

Organ samples were homogenized in bead beater tubes. Liquefied tissues were spun down at 12,000 RPM for 10 minutes at 4 °C. Resulting supernatant was filtered through a 0.22 μm filter at 12 × g RCF for 4 minutes. Cytokines were measured using a V-Plex Proinflammatory Panel1 (mouse) Kit (K15048D-1; Meso Scale Discovery) according to manufacturer’s instructions, and read on a SECTOR Imager 2400 (Meso Scale Discovery).

### Blood profiling

One hundred μl of blood samples were removed from mice at 3 and 18 hours post-injection with *B. bacteriovorus* 109J and sent to ANTECH Diagnostics (New Hyde Park, NY, USA) for blood cell profiling.

### Bacterial dissemination

To detect predatory bacterial dissemination within the host, primers targeting the 16S rRNA gene of each predatory bacterial strain were synthesized: *B. bacteriovorus* HD100 (Forward): 5′-GGAGGCAGCAGTAGGGAATA-3′, (Reverse): 5′-GCTAGGATCCCTCGTCTTACC-3′^30^; 109J (Forward): 5′-ACACGGTCCAGACTCCTACG-3′, (Reverse): 5′-ACGCTAGGATCCCTCGTCTT-3′; *M. aeruginosavorus* strain ARL-13 (Forward): 5′-GGCTTCACTTTGTCCAGAGC-3′; (Reverse): 5′-CAGAAAAACGCGAAATCCTC-3′. Samples were tested in triplicates, with each reaction containing: template (1.0 μl of cDNA synthesized above), SYBR Green PCR Master Mix (Applied Biosystems), and 500 nM (for 109J and *Micavibrio*) or 900 nM (for HD100) of each primer (synthesized at the Rutgers New Jersey Medical School Molecular Resource Facility). A 7900HT Fast Real-Time PCR System (Applied Biosystems) was used: 50 °C for 2 min (1X), 95 °C for 10 min (1X), 95 °C for 15 sec/60 °C for 1 min (40X), 95 °C for 15 sec/60 °C for 15 sec/95 °C for 15 sec (1X). For each qPCR run, a 10-fold dilution series of the standard (purified DNA from each predatory strain) was assessed in triplicate to validate qPCR performance and facilitate quantification. In addition, each qPCR run included negative controls (no template). Gene copy number was calculated using the ‘Calculator for determining the number of copies of a template,’ by URI Genomics & Sequencing Center (http://cels.uri.edu/gsc/cndna.html)^31^.

### Histology

All histopathological examination was performed by a pathologist that was not blinded to each specimen’s treatment group. Paraformaldehyde-fixed organ segments from infected mice were paraffin-embedded and stained with hematoxylin and eosin (H&E) for cellular composition as previously described^32^. Stained sections were analyzed and photographed using a Nikon Microphot-FX photomicrographic system with NIS-Elements F3.0 software (Nikon Instruments Inc, Melville, NY).

### Statistical analysis

qPCR data are presented as mean ± standard deviation. ELISA data are presented as mean ± standard error of the mean. Significant differences between the treated sample compared to respective control were examined using independent-samples student’s *t*-tests. A *P* value of <0.05 was considered significant.

## Additional Information

**How to cite this article**: Shatzkes, K. *et al.* Examining the safety of respiratory and intravenous inoculation of *Bdellovibrio bacteriovorus* and *Micavibrio aeruginosavorus* in a mouse model. *Sci. Rep.* **5**, 12899; doi: 10.1038/srep12899 (2015).

## Figures and Tables

**Table 1:** Host viability of intranasal inoculation of predatory bacteria.

Treatment	#of Mice	%Viable on Day 5	%Viable on Day 50*
Control (PBS)	5	100%	100%
*B. bacteriovorus* 109J	5	100%	100%
*B. bacteriovorus* 109J (HK)	5	100%	100%
*B. bacteriovorus* HD100	5	100%	100%
*B. bacteriovorus* HD100 (HK)	5	100%	100%
*M. aeruginosavorus* ARL-13	5	100%	100%

^*^2 mice from each treatment group from the ‘5 Day’ experiment were visually assessed for up to 50 days. HK: heat-killed.

**Table 2:** Host viability after intravenous injection of predatory bacteria.

Treatment	#of Mice	%Viable on Day 20
Control (PBS)	5	100%
– Re-inject at 10 days	5	100%
*B. bacteriovorus* 109J	5	100%
– Re-inject at 10 days	5	100%
